# Genome Re-Sequencing and Functional Analysis Places the *Phytophthora sojae* Avirulence Genes *Avr1c* and *Avr1a* in a Tandem Repeat at a Single Locus

**DOI:** 10.1371/journal.pone.0089738

**Published:** 2014-02-24

**Authors:** Ren Na, Dan Yu, B. Patrick Chapman, Yun Zhang, Kuflom Kuflu, Ryan Austin, Dinah Qutob, Jun Zhao, Yuanchao Wang, Mark Gijzen

**Affiliations:** 1 Agriculture and Agri-Food Canada, London, Canada; 2 College of Agronomy, Inner Mongolia Agricultural University, Huhhot, China; 3 College of Plant Protection, Nanjing Agricultural University, Nanjing, China; USDA ARS, United States of America

## Abstract

The aim of this work was to map and identify the *Phytophthora sojae Avr1c* gene. Progeny from a cross of *P. sojae* strains ACR10×P7076 were tested for virulence on plants carrying *Rps*1c. Results indicate that avirulence segregates as a dominant trait. We mapped the *Avr1c* locus by performing whole genome re-sequencing of composite libraries created from pooled samples. Sequence reads from avirulent (Pool1) and virulent (Pool2) samples were aligned to the reference genome and single nucleotide polymorphisms (SNP) were identified for each pool. High quality SNPs were filtered to select for positions where SNP frequency was close to expected values for each pool. Only three SNP positions fit all requirements, and these occurred in close proximity. Additional DNA markers were developed and scored in the F_2_ progeny, producing a fine genetic map that places *Avr1c* within the *Avr1a* gene cluster. Transient expression of *Avr1c* or *Avr1a* triggers cell death on *Rps*1c plants, but *Avr1c* does not trigger cell death on *Rps*1a plants. Sequence comparisons show that the RXLR effector genes *Avr1c* and *Avr1a* are closely related paralogs. Gain of virulence on *Rps*1c in *P. sojae* strain P7076 is achieved by gene deletion, but in most other strains this is accomplished by gene silencing. This work provides practical tools for crop breeding and diagnostics, as the *Rps*1c gene is widely deployed in commercial soybean cultivars.

## Introduction

Plants are challenged by a diversity of pathogens and rely on pre-existing and elaborated defense mechanisms including innate immunity. Pathogens have developed effector arsenals to successfully colonize plants and overcome plant defenses. Host plants detect microbe-associated molecular patterns (MAMPs) and pathogen effector molecules by use of immune receptors positioned on the outer plasma membrane or inside the cell cytoplasm [1]. The activation of pattern-triggered immunity (PTI) or effector-triggered immunity (ETI) leads to defense activation and pathogen containment. In many cases host immune receptors and pathogen effector proteins underlie resistance (*R*) and avirulence (*Avr*) gene interactions described by plant pathologists.

Soybean resistance to the root rot pathogen *Phytophthora sojae* is determined in part by the presence of *Rps* genes. *P. sojae* effectors that are recognized by Rps proteins and cause ETI are Avr factors. As key elements that determine disease outcome on soybean, the Avr factors of *P. sojae* have been a focus of study and targeted for identification [2]. Several *P. sojae Avr* genes have been identified to date including *Avr1a* [3], *Avr1b* [4], *Avr1d* [5], *Avr1k* [6], *Avr3a*/*5* [3], [7], *Avr3b* [8], *Avr3c* [9], and *Avr4/6* [10]. Each *Avr* gene is predicted to encode a small secreted protein containing a signal peptide followed by an RXLR (Arg-any amino acid-Leu-Arg) sequence. This conserved motif is proposed to have a role in the delivery of the effector protein to the host cell cytoplasm [11], [12]. Additional conserved features in the C-terminal or effector domains of the proteins are suggested to be important functional elements [13], [14].

The identification of *P. sojae Avr* genes has relied on map-based approaches. Segregating F_2_ populations arising from outcrosses of parental strains that differ in virulence on particular *Rps* genes have provided the basis for investigations into *Avr* determinants. This core strategy has been assisted by strain-specific associative mapping, whole genome sequence information, transcript profiling, and the annotation of some 400 predicted *P. sojae* RXLR effectors known as *Avh* genes [15]–[17]. Selecting candidate *Avh* genes *a priori* can accelerate mapping and identification but this tactic is not fail-safe. Bulked segregant analysis (BSA) or pooled sampling is a powerful method that aids *de novo* mapping by providing genetic markers linked to gene targets [18], [19]. Recently, pooling has been combined with deep sequencing technologies to rapidly map and identify target regions in a variety of organisms [20]–[22].

The objective of the present study was to identify the *P. sojae Avr1c* locus. A genetic cross of *P. sojae* strains ACR10×P7076, which differ in virulence on *Rps*1c, was employed to track the segregation of *Avr1c* in F_1_ and F_2_ progeny. Candidate *Avh* genes were selected for mapping but this was not successful, as none of the candidates co-segregated with *Avr1c*. We resorted to mapping by performing BSA and single nucleotide polymorphism (SNP) analysis of whole genome re-sequencing data. This led to the finding that *Avr1c* is part of the *Avr1a* gene cluster. The *Avr1c* gene encodes a predicted RXLR effector protein of 126 amino acids. The *Avr1a* and *Avr1c* genes are close paralogs that are nearly identical in sequence at their N-terminal regions but that have diverged substantially in their C-terminal effector domains.

## Results

### Avirulence towards *Rps*1c is a dominant trait in *P. sojae* cross ACR10×P7076

To determine how virulence towards *Rps*1c is inherited in *P. sojae*, we analyzed progeny from outcrosses between strains. The *P. sojae* strains ACR10 and P7076 differ in virulence towards soybean plants carrying the *Rps*1c gene; ACR10 is avirulent while P7076 is virulent. Both strains are virulent towards control plants that lack *Rps*1c, such as cv Williams (no known *Rps* genes) or Harosoy (*Rps*7). Outcrosses of ACR10× P7076 were performed and the resulting F_1_ and F_2_ progeny were tested for virulence towards *Rps*1c. From a total of 25 F_1_ progeny, an avirulent:virulent phenotypic ratio of 23:2 was observed. Testing of 40 F_2_ progeny derived from a single F_1_ individual resulted in consistent readouts for 28 of the progeny, whereas 12 F_2_ progeny could not be reliably scored for virulence on *Rps*1c, or had lost virulence toward control plants lacking the *Rps*1c gene. For the 28 F_2_ progeny with reliable phenotypes and that retained virulence towards the control plants, the avirulent:virulent ratio towards *Rps*1c was 22:6 (Chi-squared probability  =  0.66 for 3:1). Overall, the results suggest that avirulence towards *Rps*1c segregates as a dominant trait in outcross between *P. sojae* strains ACR10×P7076.

### Selected candidate *Avh* genes do not co-segregate with *Avr1c*

To accelerate the identification of *Avr* genes, pre-selecting candidate genes has proven a successful tactic in many cases [5], [8], [9], [23]–[25]. Briefly, candidates are chosen based upon sequence characteristics, expression and polymorphism data and tested for co-segregation or association with the virulence trait, or directly screened for functional interaction with selected *R*-genes. We selected a total of 24 *Avh* genes as candidates for *Avr1c*, based upon comparison of *Avh* gene sequences in the reference *P. sojae* strain P6497 and re-sequenced strains P7064, P7074 and P7076, as shown in Supplementary Table S1. Strains P6497, P7064, and P7074 are avirulent, while strain P7076 is virulent on *Rps*1c. Cleaved amplified polymorphic (CAP) markers were designed to score each of the 24 candidate *Avh* genes in the ACR10×P7076 F_2_ progeny. Unfortunately, none of the 24 candidate *Avh* genes tested co-segregated with *Avr1c*, so this approach was not successful.

### Deep sequencing of progeny pools identifies SNPs linked to *Avr1c*

**Figure 1:**
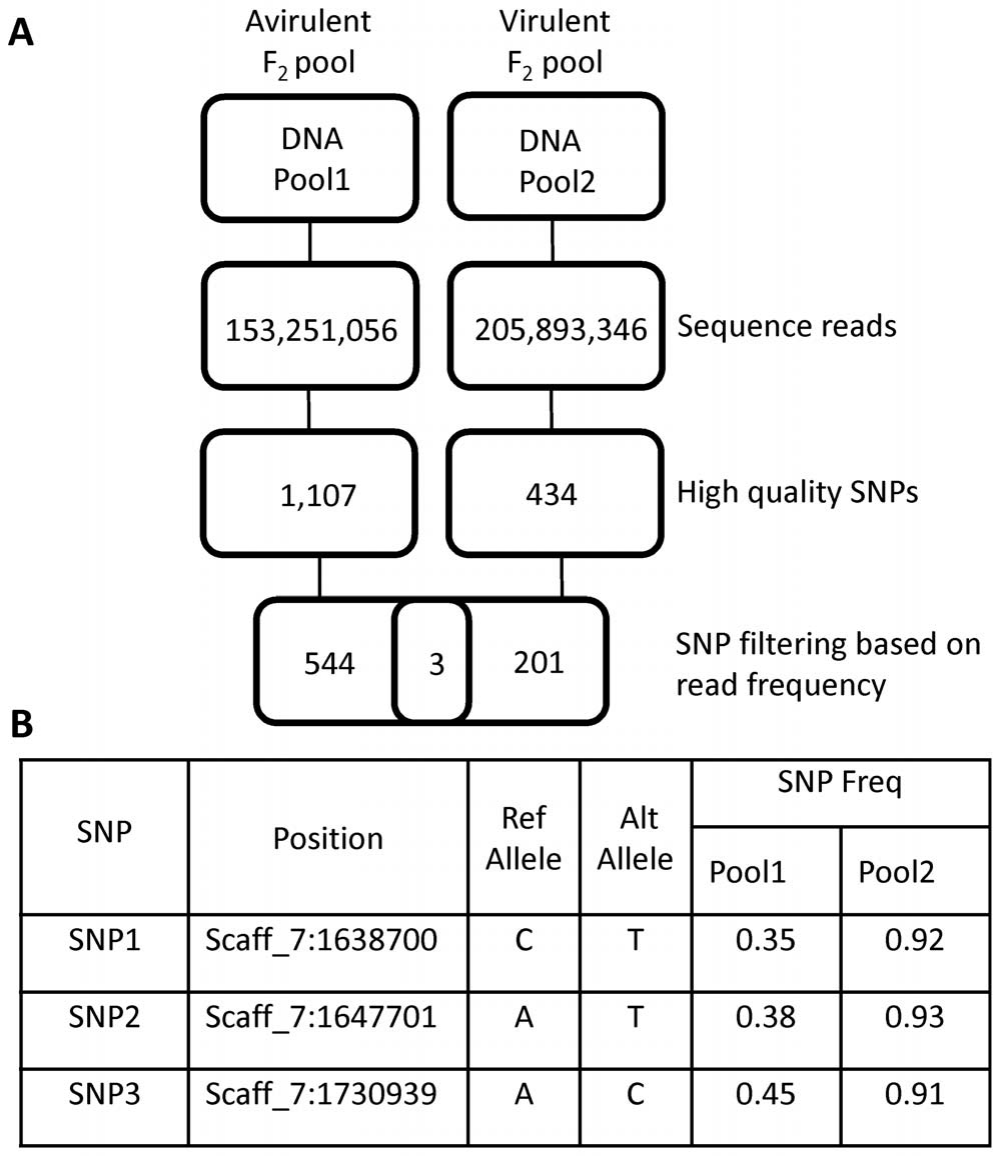
Identification of SNPs linked to *Avr1c* by bulked segregant analysis and deep sequencing. **A,** The procedure for discovery of candidate SNPs linked to *Avr1c* is shown. Selected F_2_ progeny from a cross of *P. sojae* strains ACR10×P7076 were pooled according to their virulence phenotype. The avirulent (A) Pool1 and virulent (V) Pool2 composite DNA samples were deeply sequenced. Sequence reads were aligned against the reference genome, and SNPs were identified and filtered based upon quality scores. High quality SNPs were further filtered according to the predicted SNP frequencies for Pool1 and Pool2. After processing, only three SNPs passed all requirements. **B,** Genome location of three candidate SNPs. These three candidate SNPs occur in close physical proximity in the reference genome assembly. All three sites fall within a 92 kb segment on Scaffold_7 (V5.0). Reference (Ref) allele and alternate (Alt) alleles for three SNPs are shown. The SNP frequencies (Freq) in each pool are also shown.

The *de novo* mapping of *Avr1c* was accomplished using next-generation sequencing and BSA applied to two pools of F_2_ progeny [18], [20], [21]. Equal amounts of DNA from six avirulent (Pool1) or six virulent (Pool2) F_2_ individuals were combined to construct two composite genomic libraries for deep sequencing and SNP analysis. In excess of 150 million sequence reads were obtained from each of the pools, as detailed in Table 1 and Figure 1. Reads were aligned to the reference genome P6497 and SNPs called from the resulting alignment map. Mapping SNPs linked to *Avr1c* proceeded under the following assumptions. Based on the dominant phenotype observed for *Avr1c*, the avirulent pool (Pool1) was expected to contain both alleles of *Avr1c* and therefore consist of a mix of homozygous dominant and heterozygous genotypes in a ratio of 1:2. The expected non-reference base allele frequency for a causal SNP in this pool should then be the average of one homozygous dominant allele (0) and two heterozygous alleles (0.5) for an expected frequency of 0.33. However, the virulent pool (Pool2) should consist of exclusively homozygous recessive genotypes with only the virulent allele of *Avr1c* represented and thus possess a non-reference base allele frequency of 1.0 for the causal SNP. These numbers may vary slightly due to effects in sampling, sequencing errors or mapping accuracy. However, both the causal SNP for virulence as well as nearby SNPs genetically linked to the loci should exhibit allele frequencies very close to these expectations. Conversely, for SNPs linked to the avirulent allele, the reciprocal values of 0.67 (Pool1) and 0.0 (Pool2) would be expected. Therefore, our causal SNP and those linked to it should be identifiable by selecting for SNPs that exhibit the expected allele frequency in each respective pool.

**Table 1:** Whole genome re-sequencing of composite and strain specific *P. sojae* libraries.

Sample	Total reads^1^ (millions)	Q30^2^ (%)	Mapped reads^3^ (%)	Average Coverage^4^
Pool1	153.3	84.3	95.9	180
Pool2	205.9	84.1	95.8	241
ACR10	164.0	89.0	97.6	196
P7076	191.7	87.2	98.3	230

1 Illumina HiSeq 2000, read length 100 bp.

2 Sequence reads with quality score >30.

3 Sequence reads that map to the 82 Mb *P. sojae* reference genome assembly.

4 Average depth of sequence read coverage.

With this is mind, a sequence alignment map (SAM) was generated by aligning reads from each sequenced pool to the P6497 reference. The SNPs were then called using Samtools and filtered for quality (Phred quality > 30). A total of 1,107 (Pool1) and 434 (Pool2) high quality SNPs were obtained and filtered using a window of acceptable allele frequencies based on the expected values for each pool. A window of acceptable non-reference allele frequency values ranging from 0.18 to 0.48 was applied to Pool1 SNPs, and a window of 0.9 to 1.0 was used to select for Pool2 SNPs. The reciprocal values were also used in order to capture SNPs linked to either allele. By this criteria, 547 (Pool1) and 204 (Pool2) SNP positions were found, but only three SNPs exhibited allele frequencies that fit both pool expectations, as shown in Figure 1A. Remarkably, as would be expected from genetic linkage, all three of the identified SNPs occur in close physical proximity within a 92 kb segment on Scaffold_7 in the *P. sojae* reference genome assembly (v5.0) (Figure 1B).

### *Avr1c* is located in the *Avr1a* gene cluster

**Figure 2:**
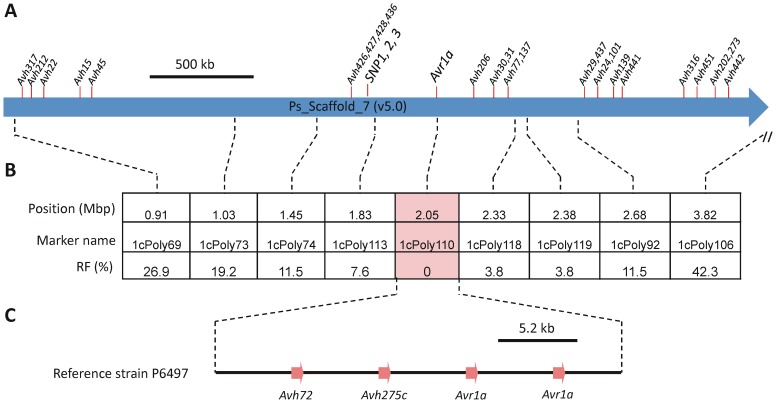
Genetic and physical mapping of *Avr1c* region. **A,** Physical map of *Avr1c* region. The position of the three identified SNP markers, *Avr1a*, and 25 predicted *Avh* genes are shown. **B,** Genetic analysis of the *Avr1c* region. The position of DNA markers and their recombination frequency (RF%) with *Avr1c* in a segregating F_2_ population (n = 28) is indicated; Mbp, mega base pair. **C,** Predicted arrangement of the *Avr1a* locus in *P. sojae* reference strain P6497.

The SNPs identified in our mapping occurred in close proximity in a region rich in *Avh* genes, including the previously identified *Avr1a* locus, as shown in Figure 2A. In order to verify that *Avr1c* occurs in this genome region and to more accurately map its position, a set of DNA markers was developed based upon polymorphic sites identified by comparison of the *P. sojae* reference genome and sequence reads from each of the pools. Useful markers were also available from previous mapping work on *Avr1a* [3], [26]. Each marker was scored in a mapping population of 28 F_2_ progeny. The results indicate that *Avr1c* maps precisely to the *Avr1a* locus (Figure 2B).

### The predicted RXLR effector gene *Avh275c* corresponds to *Avr1c*

Previous work on *Avr1a* indicated that this locus is highly polymorphic and displays copy number variation among *P. sojae* strains [3]. The reference strain P6497 contains a tandem array of four related 5.2 kb segments, as shown in Figure 2C. Two of these segments include copies of *Avr1a* while adjacent segments include paralogous genes named *Avh275c* and *Avh72*. The *Avh72* open reading frame includes a frame-shift and no transcripts can be detected for this gene, indicating that *Avh72* is a pseudogene. However, the *Avh275c* open reading frame is intact and transcripts can be detected for this gene in several *P. sojae* strains. The *Avh275c* gene encodes a predicted RXLR effector protein of 126 amino acids. Results described below indicate that *Avh275c* corresponds to *Avr1c*, thus for simplicity, we will hereafter refer to the *Avh275c* gene as *Avr1c*.

**Figure 3:**
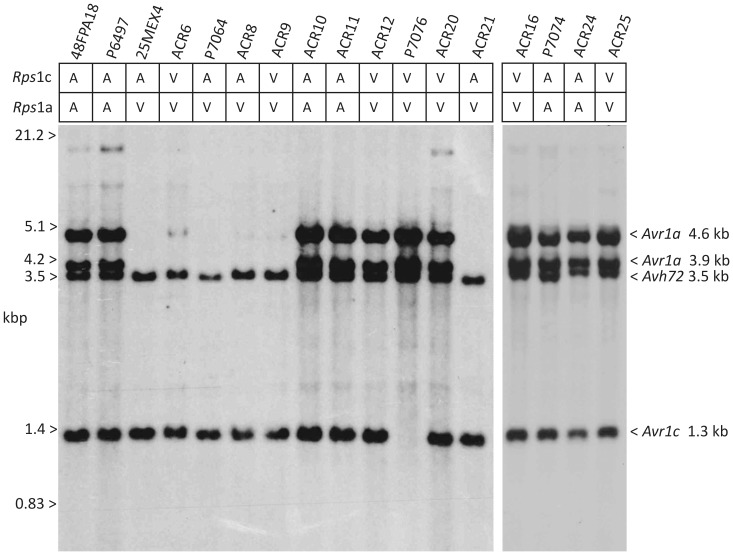
Genomic DNA blot hybridization showing *P. sojae* strain-specific deletions of *Avr1a* and *Avr1c*. Selected strains of *P. sojae* genomic DNA were digested with *Pml*I and separated by electrophoresis prior to blotting and hybridization. Virulence phenotype of the *P. sojae* strains on *Rps*1c or *Rps*1a plants is shown as virulent (V) or avirulent (A). The positions and sizes of the *Pml*I segments of *Avr1a*, *Avr1c*, and *Avh72* genes are indicated on the right. The sizes of DNA markers are shown on the left; kb, kilo base pair. The *Avr1c* gene is deleted from parental strain P7076.

**Figure 4:**
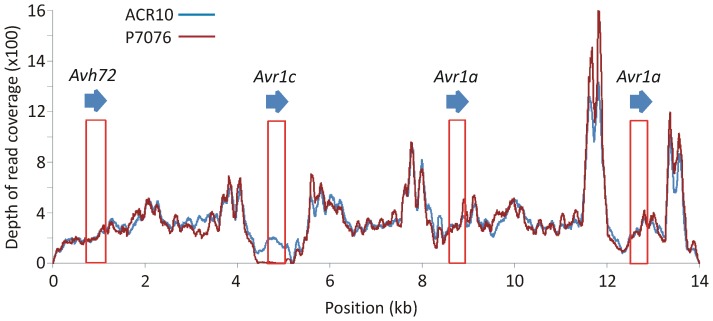
Sequence read coverage of the *Avr1a/Avr1c* region from re-sequencing of *P. sojae* strains ACR10 and P7076. An assembly of the *Avr1a* region from reference strain P6497 was used to align sequence reads from parental strains ACR10 and P7076. The DNA segment corresponding to the *Avr1c* gene lacks sequence reads in parental strain P7076. The longest segment of identity between the *Avr1a* and *Avr1c* DNA sequences is 98 nucleotides, which is shorter than the read length of 100 nucleotides.

To investigate the structure of the *Avr1c* locus among *P. sojae* strains, genomic DNA was digested with the restriction enzyme *Pml*I, blotted, and hybridized with a probe that detects *Avh72*, *Avr1c* and *Avr1a.* The *Pml*I digestion is known to resolve each of the four copies in the tandem array, including *Avh72*, *Avr1c*, and the two copies of *Avr1a*, as we have previously demonstrated [3]. The results shown in Figure 3 indicate that *P. sojae* strain P7076 lacks a copy of the *Avr1c* gene, but that all other strains tested possess a copy of *Avr1c*. Additionally, data from re-sequencing of the parental strains ACR10 and P7076 was used to measure read coverage across the *Avr1c* segment, as constructed for the reference strain P6497 [3]. As is shown in Figure 4, there is no coverage in the *Avr1c* region in virulent strain P7076, while this region is covered by sequence reads in the avirulent strain ACR10. Thus, the DNA blot and the sequence coverage analyses indicate that *Avr1c* is deleted from *P. sojae* strain P7076 but that copies of *Avh72* and *Avr1a* remain in this tandem gene cluster.

**Figure 5:**
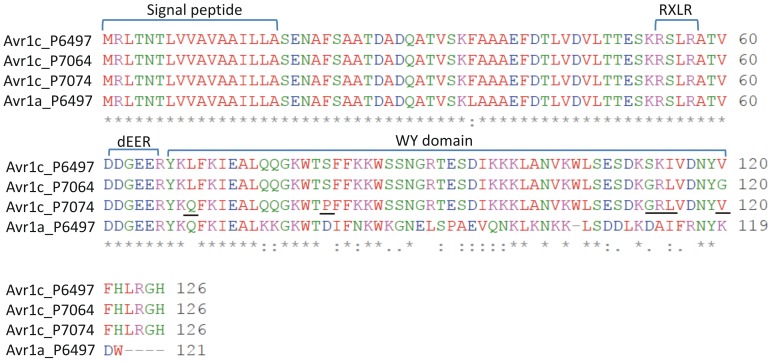
Amino acid sequence alignment of the predicted proteins for Avr1a and each of the three alleles of Avr1c. The residues are colored according to their physicochemical properties. Signal peptide, RXLR and dEER motifs, and WY-domain are shown, and polymorphic residues among the three Avr1c alleles are underlined. An asterisk (*) indicates positions which have a single, fully conserved residue; a colon (:) indicates conservation between groups of strongly similar properties; a period (.) indicates conservation between groups of weakly similar properties.

Sequence analysis revealed three different *Avr1c* alleles within our *P. sojae* strain collection (Figure S1). These include the *Avr1c-1* allele (represented by strain P6497; also present in strain ACR10), the *Avr1c-2* allele (represented by strain P7064) and the *Avr1c-3* allele (strain P7074). The *Avr1a* DNA sequence encoding the open reading frame for the protein is not polymorphic among known strains of *P. sojae* that carry copies of the gene. Comparison of the predicted amino acid sequences of Avr1a and each of the Avr1c alleles is shown in Figure 5. The N-terminal portions of the Avr1a and Avr1c predicted proteins are nearly identical, while the C-terminal regions have diverged substantially. Among the Avr1c alleles, Avr1c^P7064^ displays four amino acid changes (S113G, K114R, I115L,V120G) and Avr1c^P7074^ displays five changes (L69Q, S82P, S113G, K114R, I115L), compared to the reference allele Avr1c^P6497^.

**Figure 6:**
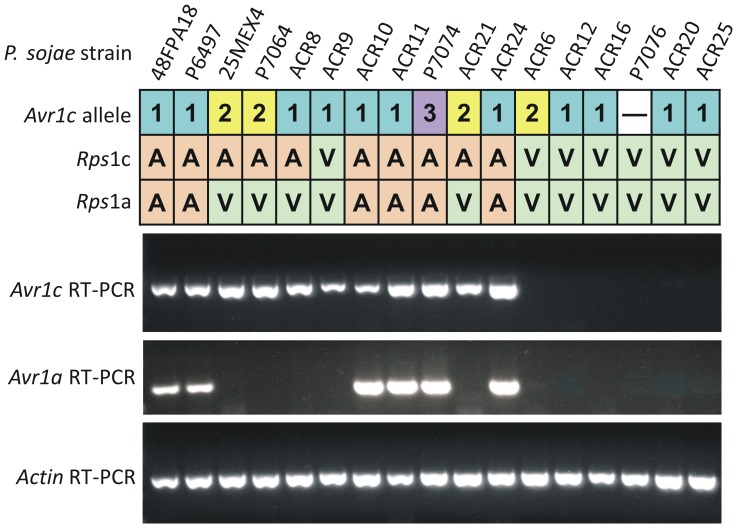
Analysis of alleles of *Avr1c*, and transcripts of *Avr1c* and *Avr1a* in *P. sojae* strains. Shown at the top of the figure are the *Avr1c* alleles present in each of the selected *P. sojae* strains. Virulence phenotype of the *P. sojae* strains on *Rps*1c or *Rps*1a plants is shown as virulent (V) or avirulent (A). Reverse transcriptase polymerase chain reaction (RT-PCR) analysis was performed using mRNA from mycelia cultures, and *Avr1a* and *Avr1c* specific primers, to test for transcripts of these two genes. Results from RT-PCR of the control gene *Actin* are also shown.

To test for the presence of *Avr1c* mRNA transcripts among *P. sojae* strains, we performed reverse transcriptase polymerase chain reaction (RT-PCR). The *Avr1a* transcript was also measured for comparison since these two genes occur adjacent to each other. Results show that transcripts of *Avr1c* and *Avr1a* are present in strains that are avirulent towards *Rps*1c and *Rps*1a, respectively, but not in the corresponding virulent strains. One exception is strain ACR9, which expresses the *Avr1c* transcript but nonetheless is virulent on *Rps*1c (Figure 6).

### Transient expression of *Avr1c* triggers cell death in *Rps*1c plants

**Figure 7:**
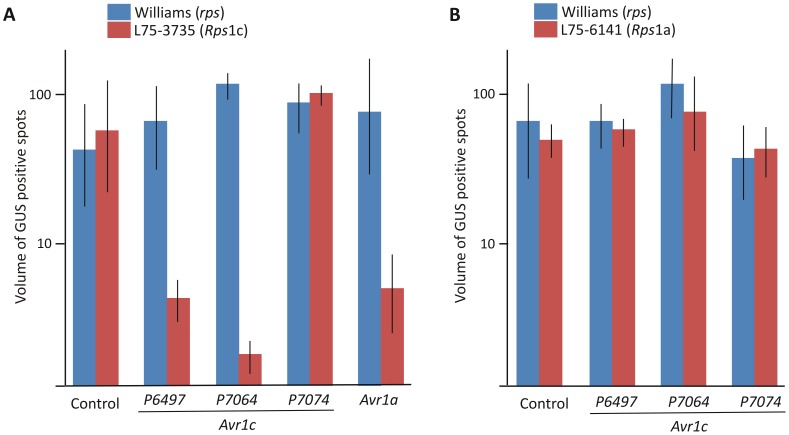
Transient expression of *Avr1c* and *Avr1a* triggers cell death in *Rps*1c soybean plants. Measurement of cell death in soybean leaves by co-bombardment and transient expression of a GUS reporter together with a test gene. Test genes that cause cell death reduce GUS expression and blue staining. **A,** Leaves of Williams (*rps*) and the isoline L75-3735 (*Rps*1c) tested with each of the three *Avr1c* alleles, and *Avr1a*. **B,** Leaves of Williams (*rps*) and the isoline L75-6141 (*Rps*1a) tested with each of the three *Avr1c* alleles. Control test gene in each experiment corresponds to a synthetic *Avr1a* sequence with a frame-shift mutation. Results show means and standard errors of three independent biological replicates, with a minimum of three leaves per treatment, per replicate.

**Figure 8:**
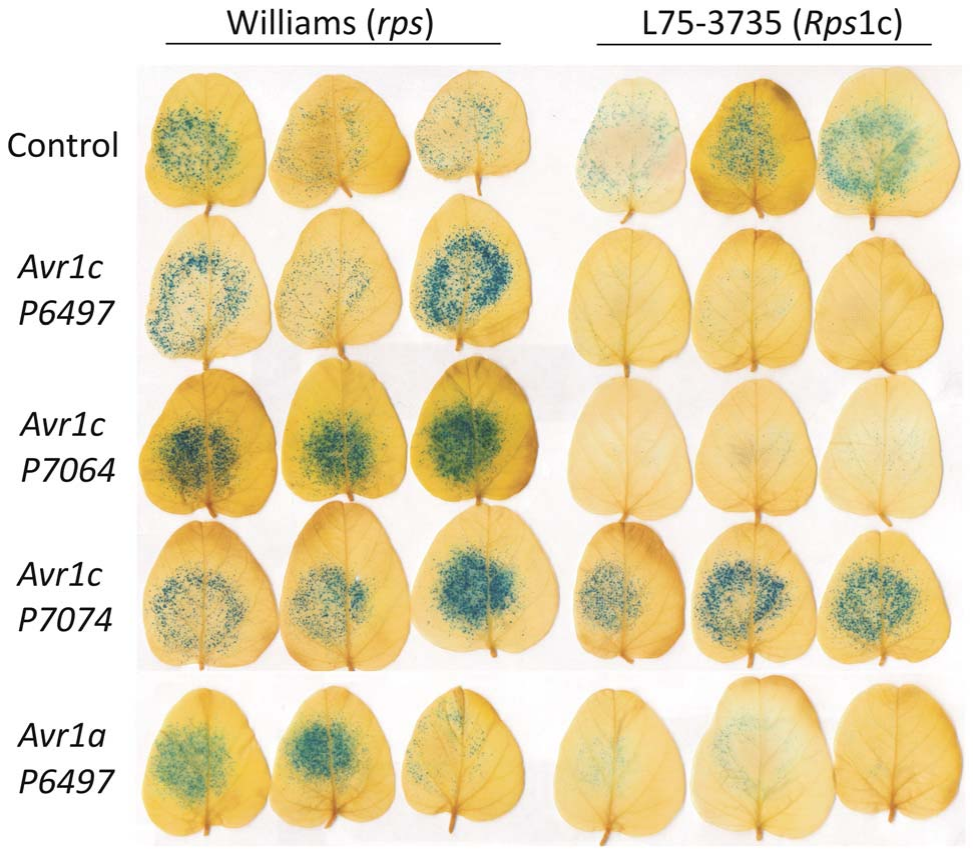
Photographs of leaves of Williams (*rps*) and the isoline L75-3735 (*Rps1c*) tested with each of the three *Avr1c* alleles, and *Avr1a*. Results from a representative co-bombardment experiment are shown, after staining for GUS expression.

**Figure 9:**
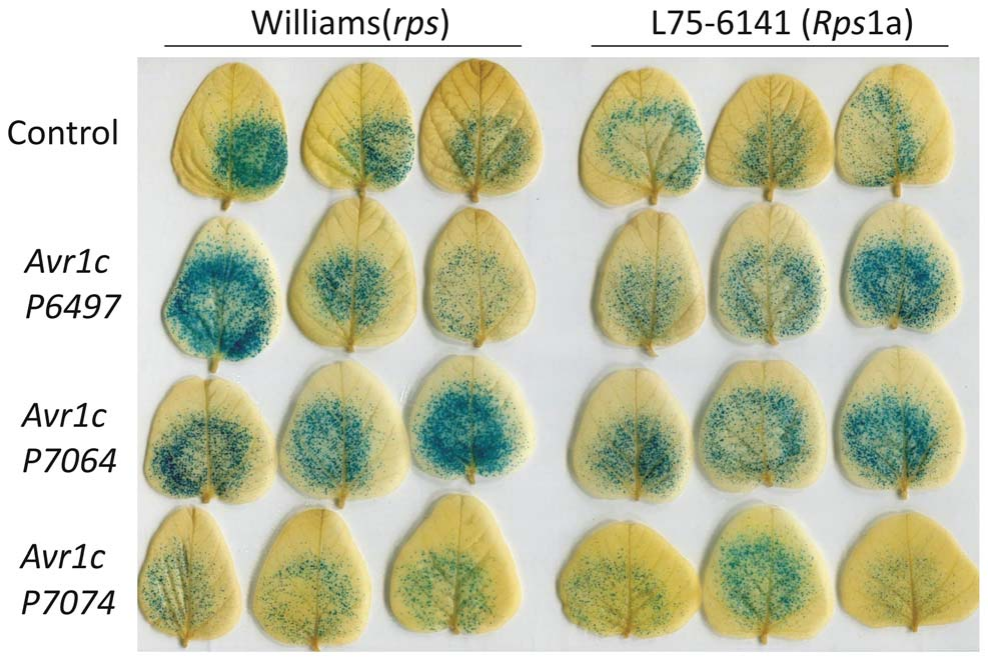
Photographs of leaves of Williams (*rps*) and the isoline L75-6141 (*Rps*1a) tested with each of the three *Avr1c* alleles. Results from a representative co-bombardment experiment are shown, after staining for GUS expression.

Transient expression of an Avr effector protein in a soybean plant containing a matching *Rps* gene triggers cell death. In order to test whether Avr1c can trigger cell death in an *Rps*1c soybean plant, transient expression of *Avr1c* together with a reporter gene was performed in a co-bombardment assay [27]. Plasmid constructs of *Avr1c* excluding the native signal peptide sequence, were bombarded into *Rps*1c soybean leaves along with glucuronidase (GUS) reporter gene to measure cell death. This was done for each of the three *Avr1c* alleles in the soybean cv Williams (no known *Rps* genes) and the isoline L75-3735 (*Rps*1c). Expression of *Avr1c^P6497^* and *Avr1c^P7064^* triggered cell death specifically in leaves of *Rps*1c plants, whereas *Avr1c^P7074^* did not trigger cell death in *Rps*1c plants, as shown in Figure 7 and Figure 8. None of the alleles triggered cell death in control plants lacking the *Rps*1c gene. Results from this functional assay indicate that Avr1c^P6497^ and Avr1c^P7064^, but not Avr1c^P7074^, are recognized by Rps1c. We also tested the interactions between *Avr1a* and *Rps*1c, and for each of the *Avr1c* alleles and *Rps*1a, since *Avr1c* and *Avr1a* share considerable sequence identity. Results indicate that expression of *Avr1a* triggers cell death in *Rps*1c leaves, but that none of the *Avr1c* alleles trigger cell death in *Rps*1a leaves, as determined by the co-bombardment assay (Figures 7, 8, 9).

## Discussion

The soybean *Rps*1c gene is extensively deployed for the management of root and stem rot disease caused by *P. sojae*. Based on recent variety trial data shown in Table 2, *Rps*1c is the most prevalent source of resistance to *P. sojae* in the province of Ontario, where over 1 million hectares of land is annually dedicated to soybean production. The *Rps*1c gene is also widely deployed in the USA and other major soybean growing regions. Thus, identification of the *Avr1c* gene in *P. sojae* will have practical uses in soybean breeding and pathogen diagnostics.

**Table 2:** *Rps*1c is the most prevalent known source of *P. sojae* resistance in soybean lines entered in Ontario variety trials^1^.

*Rps* gene(s)	Number of lines
Not known/undisclosed	137
*Rps*1c	53
*Rps*1k	36
*Rps*3a	6
*Rps*1a	5
*Rps*1c + *Rps*3a	3
*Rps*1c + *Rps*1k	1

1 Source: 2012 Ontario Soybean Variety Trials. Data collected 2010–2012. Conducted by the Ontario Oil and Protein Seed Crop Committee www.gosoy.ca.

Genomic resources and annotation of RXLR effector repertoires for oomycete plant pathogens have facilitated *Avr* gene identification in several species. Large scale effector screens have proven to be successful for identifying or predicting *Avr* determinants in *Phytophthora infestans* [24], [28] and *Bremia lactucae* [29], but this method is less useful in *P. sojae* because of the difficulty in performing *Agrobacterium* mediated infiltration and transient expression in soybean. However, being a homothallic organism *P. sojae* offers certain advantages over predominately heterothallic species such as *P. infestans* and *B. lactucae*. For example, creating F_2_ populations for genetic mapping is generally possible for homothallic but not heterothallic species. In fact, identification of all known *P. sojae Avr* genes has relied on mapping in segregating F_2_ populations. Most recently, this strategy has been accelerated by pre-selection of candidate RXLR effectors [5], [8], [9]. The failure of the candidate gene approach in the present study was primarily a result of an incomplete RXLR inventory. To select candidates based on strain-specific polymorphisms, we relied on previous work that described a comparative genome-wide analysis of *Avh* genes among *P. sojae* strains [15]. Unfortunately, *Avh275c* was not included in the gene set because of poor annotation of tandem arrays in the reference genome. In addition to *Avh275c*, we also should have included known *P. sojae Avr* genes (or closely linked markers) in our initial screen, as this would have revealed co-segregation of *Avr1c* and *Avr1a*.

Of course, it is easier to select candidates in hindsight, and any approach of choosing genes *a priori* will always carry a risk because it involves uncertainties. In contrast, *de novo* mapping of genes represents an unbiased and more robust method for target identification but can involve a greater research investment. Deep sequencing technologies combined with bioinformatics offer powerful tools for gene mapping. Previously, BSA has been combined with amplified fragment length polymorphism (AFLP) [26], and microarray analyses [3] for mapping *Avr* genes in *P. sojae*. Our present scheme of combining BSA with whole genome re-sequencing is a natural step forward, especially since this technique has proven successful in other organisms [20], [22]. With a high quality reference genome assembly, the path from finding linked markers to identifying target genes can proceed rapidly, as we have shown in this study.

Our conclusion that avirulence towards *Rps*1c is inherited as a dominant trait in crosses between *P. sojae* strains ACR10×P7076 is consistent with the segregation results, despite the deviations that were observed. Previous inheritance studies have suggested that *Avr1c* is dominant [30] or recessive [31], [32], but the strains used in past studies were different from those used in the present analysis. We show that the *Avr1c* gene is deleted from the *Rps*1c-virulent strain, P7076, which is consistent with avirulence being dominant in the cross ACR10×P7076. Our finding that many *P. sojae* strains carry a copy of *Avr1c*, yet do not express the gene, indicates that gain of virulence on *Rps*1c can also result from gene silencing. This could also explain past inheritance results that showed virulence towards *Rps*1c to be dominant in F_1_ hybrids [32], since it is known that gene silencing in *P. sojae* can cause transgenerational epigenetic inheritance that results in gain of virulence [33], [34]. In fact, comparison of results of RT-PCR analyses suggests transcriptional switching of gene expression/silencing of *Avr1c* and *Avr1a* in clonally propagated *P. sojae* cultures, because we noted some differences in expression between a previous study [3] and the present one, for strains ACR6, ACR9, ACR16, and ACR20. Variation in virulence of clonal *P. sojae* and *P. infestans* cultures is well known [35], [36]. The alternate loss and recovery of virulence in successive clonal generations has puzzled investigators [35], but our results indicate that epiallelic switching of effector gene expression is a mechanism that could account for this phenomenon.

Besides gene deletion and gene silencing, our results suggest that *P. sojae* can escape *Rps*1c-mediated immunity by other means. The transient expression assay indicates that Avr1c^P6497^, Avr1c^P7064^, and Avr1a are recognized by *Rps*1c plants but that Avr1c^P7074^ is not. There are five amino acid polymorphisms between Avr1c^P6497^ and Avr1c^P7074^, but three of these (S113G, K114R, I115L) are shared with Avr1c^P7064^, and one (L69Q) with Avr1a. Thus, we hypothesize that the critical change to Avr1c^P7074^ that enables it to escape Rps1c is the S82P mutation. Introduction of a proline residue represents a severe change with potential to alter the secondary structure in a region that is predicted to be alpha-helical, providing a rationale for the importance of the S82P mutation. However, *P. sojae* strain P7074, which carries and expresses the *Avr1c^P7074^* allele, is avirulent towards *Rps*1c soybean plants. We propose that expression of *Avr1a* by strain P7074 triggers immunity on *Rps*1c, because our results show that Rps1c can detect Avr1a in addition to Avr1c^P6497^ and Avr1c^P7064^. We predict that a *P. sojae* strain expressing the *Avr1c^P7074^* allele but lacking *Avr1a* transcripts will be virulent towards *Rps*1c, but this remains to be demonstrated.

Yet another mechanism to evade *Rps*1c immunity must be invoked to account for the finding that strain ACR9 is virulent on *Rps*1c plants. Results shown here indicate that ACR9 expresses transcripts corresponding to the *Avr1c^P6497^* allele, which should trigger immunity on *Rps*1c based on the transient expression results, yet ACR9 is virulent on *Rps*1c plants. Each of these tests, mRNA analysis and phenotypic scoring, was replicated several times during the course of the present study with consistent results. Additionally, plants inoculated with strain ACR9 tested positive for *Avr1c* expression by RT-PCR (not shown). Given the results, how do we explain the virulence of ACR9 on *Rps*1c plants? A possible explanation is that there are additional effectors in strain ACR9 that suppress ETI caused by the Avr1c-Rps1c interaction. It is known that particular RXLR effectors can suppress immune responses triggered by other RXLR effectors, and that potential *Avr-R* gene interactions can be masked by these apparent epistatic effects [15], [37]–[41].

Epistatic interactions could also cause unusual inheritance behaviour, especially if combined with gene-conversion or mitotic crossing over events that commonly occur in hybrids from outcrosses of *P. sojae* [42] and other species of *Phytophthora* [43]. Thus, it is possible that the phenotypic penetrance or inheritance of *Avr1c* in particular hybrid progeny may be influenced by epistatic loci, gene conversion, and mitotic crossing over. This is in addition to the epigenetic affects discussed earlier.

Our past work on *Avr1a* demonstrated that gain of virulence on *Rps*1a is caused by gene deletion or gene silencing [3]. In contrast to *Avr1c*, no sequence polymorphisms within the *Avr1a* open reading frame were detected among the *P. sojae* strains that carry copies of *Avr1a*. It is also evident that *Avr1c* and *Avr1a* are individually and collectively dispensable, as are most known *Avr* genes in *P. sojae*.

The transient expression assays suggest that Rps1c can recognize Avr1c and Avr1a. This conclusion will require further work to verify, such as by performing transformation of *P. sojae* to demonstrate that avirulence to *Rps*1a and *Rps*1c can be acquired by ectopic expression of *Avr1a*. Another example of an apparent dual specificity of a soybean *Rps* gene was reported recently, specifically that *Avr1b* and *Avr1k* are both capable of triggering *Rps*1k mediated immunity [6]. However, the caveat is that these suggestions are based upon genetic isolines that have been developed for the various *Rps* genes. Soybean *Rps* genes remain poorly characterized at the molecular level but it is known that the *Rps*1 locus consists of a large cluster of nucleotide-binding, leucine-rich repeat type of immune receptor genes [44], [45]. Thus, there are alternative possibilities to account for the apparent dual specificity of *Rps*1k and *Rps*1c, such as separate but closely linked *R*-genes that recognize each of the *Avr* factors individually. Indeed, the variety trial data presented in Table 2 suggests the existence of a soybean line that possess both *Rps*1k and *Rps*1c, an occurrence that would be unlikely if these two genes are truly allelic.

**Figure 10:**
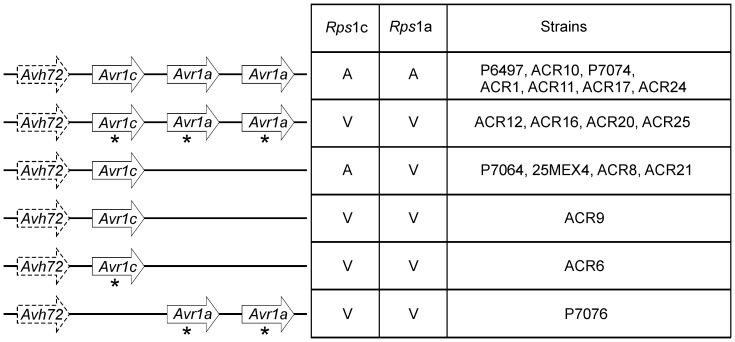
A model for the structure and expression of the *Avr1a/1c* locus in *P. sojae* strains. Results from the present study are summarized in this illustration of the *Avr1a/1c* locus. Bold arrows indicate predicted genes; *Avh72* is shown with a dashed line because this is predicted to be a pseudogene due to a frame shift mutation. For *Avr1c* and *Avr1a*, an asterisk (*) indicates gene-silencing. Disease outcome on *Rps*1c and *Rps*1a soybean plants is shown: avirulent (A); or virulent (V). Gene silencing or gene deletion can account for gain of virulence in the *P. sojae* strains shown here, except for strain ACR9. It is not known how ACR9 evades *Rps*1c recognition, but an epistatic effector that suppresses the *Avr1c*-*Rps*1c interaction could explain the result.

A model for the structure and expression of the *Avr1a/1c* locus in different strains of *P. sojae* is presented in Figure 10. We acknowledge that our results present uncertainties with regard to this model, but we feel that sufficient evidence supports our conclusion that *Avr1c* occurs in the *Avr1a* gene cluster and corresponds to the RXLR effector *Avh275c*. These findings have practical importance in soybean breeding and disease management, especially since *Rps*1c is among the most widely deployed sources of *P. sojae* resistance presently in use.

## Materials and Methods

### Plant materials, *Phytophthora sojae* strains and virulence scoring

Soybean (*Glycine max*) cultivar Williams (*rps*) and the corresponding isolines L75-3735 (*Rps*1c), L75-6141 (*Rps*1a) from the collection at Agriculture and Agri-Food Canada were used to evaluate the virulence of pathogen cultures and for transient expression by particle bombardment. The cultivar Harosoy (*Rps*7) and the corresponding isolines Haro14 (*Rps*1c) Harosoy 63 (*Rps*1a) were also used to replicate the experiments in a separate genetic background. For virulence assays, 10 soybean seeds were sown in 10 cm pots containing soil-less mix (Pro-Mix ‘BX’, Premier Horticulture Ltd, Riviere-du-Loup, Canada). A total of three pots per isolate were tested for each replicate, and a minimum of three independent biological replicates were performed for each *P. sojae* culture tested. Pots were watered with 3 mg/L 20-20-20 (N-P-K) fertilizer. Plants were grown in a controlled growth chamber with supplemental light (16 h photoperiod with 25°C day and 16°C night temperatures) for one week before inoculations.

*Phytophthora sojae* isolates were from the collection at Agriculture and Agri-Food Canada, London, ON. The origin of each strain has been described [3], [7]. Performing sexual crosses of *P. sojae* strains ACR10×P7076 and the generation of F_1_ and F_2_ progeny has been described [5], [33]. All of the isolates were routinely maintained on 2.5% (v/v) vegetable (V8) juice medium at 25°C in the dark. The *P. sojae* cultures were transferred to 0.9% (v/v) V8 agar plates for 5 to 7 days prior to green plant inoculations. Methods of hypocotyl inoculation and virulence scoring have been described [3].

### Nucleic acid isolation, RT-PCR and DNA blot hybridization

The purification of RNA from *P. sojae* mycelium and transcript analysis by reverse transcriptase PCR (RT-PCR) was carried out as previously described [46]. Total RNA was treated with DNAse I (Invitrogen). The RT-PCR was conducted on 1 µg RNA using a reverse transcriptase (SuperScriptIII, Invitrogen) system according to the manufacturer’s instructions. Genomic DNA from *P.sojae* mycelium was isolated by phenol chloroform extraction and ethanol precipitation. Genomic PCR amplifications were performed using 15 ng DNA, 0.5 µM primers, 0.25 mM dNTPs, Taq polymerase and supplied buffer. The following PCR program was used: 94°C for 2 min, 40 cycles of 94°C for 40 s, 58°C for 40 s, 72°C for 30 to 60 s (varied with the PCR product size) and a final extension of 72°C for 10 min. The *P. sojae actin* gene was used as a control.

Genomic DNA isolation from *P. sojae* mycelial cultures, digestion with the restriction enzyme *Pml*I, electrophoretic separation, blotting and hybridization was performed according to standard protocols [47]. For hybridization, a DIG-labeled probe corresponding to the full length *Avr1a* open reading frame was prepared. This probe hybridizes to *Avr1a*, *Avr1c*, and *Avh72*.

### Transient expression assays

Soybean plants were grown in the growth chambers as described above. Leaves from 14 d old plants were harvested for co-bombardment. Primers used for cloning *Avr1c* segments into the transient expression vector pFF19 are provided in Supplementary Table S2. The three different *Avr1c* open reading frames (Avh275c^P6497^, Avh275c^P7064^ and Avh275c^P7074^) and the *Avr1a* open reading frame were amplified using specific primers and cloned into the 35S promoter-derived plant expression vector pFF19 using *Bam*HI/*Pst*I restriction sites. The signal peptides were omitted from all constructs. Soybean leaves were transformed by co-bombardment with plasmids encoding a glucuronidase (GUS) reporter gene and a test construct, using tungsten beads. Co-bombardment assays were performed as previously described [48]. For the control, the test gene corresponded to a frame-shift mutant of *Avr1a*. Leaves were washed in 70% ethanol and photographed using a digital camera. Sample images were obtained and processed, and the volume of GUS-positive spots was calculated as the intensity (measured in absorbance units) multiplied by the area (mm^2^) [5].

### Deep sequencing and SNP analysis

The *P. sojae* parental strains ACR10 and P7076 and a total of 12 F_2_ individuals from the ACR10×P7076 cross were selected, including six representing each virulence phenotype towards soybean *Rps*1c plants. The F_2_ cultures were used for creating Pool1 (avirulent) and Pool2 (virulent). Cultures were grown and DNA purified, as described above, for each of the 12 F_2_ cultures and two parents. Equal amounts of DNA were combined to create composite samples of Pool1 and Pool2. Library construction and deep sequencing (Illumina HiSeq 2000) of ACR10, P7076, Pool1 and Pool2 was performed by The Centre for Applied Genomics (Sick Kids Hospital, Toronto, Canada). In excess of 150 million sequence reads were obtained for each sample.

The sequence reads from the two pools were trimmed and aligned with the *P. sojae* reference stain P6497 V3.0 assembly downloaded from the DOE-JGI website. Specifically the unmasked assembly was accessed on January 16 2012 at:

http://genome.jgi.doe.gov/Physo3/download/Physo3_AssemblyScaffolds.fasta.gz.

Sequence reads were mapped using the Burrows Wheeler Alignment (BWA) version 0.6.1 to generate a Sequence Alignment Map (SAM) [49], [50]. The SAM-tools version 0.1.18 was used to call SNPs to a binary variant call format (BCF) file which was filtered for SNP quality using vcfutils.pl from the Samtools package. Allele frequencies specific to the non-reference base for each SNP were calculated from the DP4 values in the BCF file.

Amino acid alignment was performed using the ClustalW algorithm (http://www.ebi.ac.uk/Tools/msa/clustalw2/). The SignalP 4.0 server was used for secretion signal peptide prediction (http://www.cbs.dtu.dk/services/SignalP/).

### Data deposition

The *Avr1c* sequences described in this study were deposited to NCBI GenBank, accession numbers KF661323, KF661324, and KF661325. Genome re-sequencing data for *P. sojae* strains ACR10 and P7076 is available at NCBI Sequence Read Archive, BioProject accession PRJNA230486.

## Supporting Information

Figure S1 **Alignment of DNA sequences of** ***Avr1c*** **and** ***Avr1a*** **.**
(PDF)

Table S1 **A list of 24 candidate** ***Avh*** **genes tested for co-segregation with** ***Avr1c*** **in the** ***P. sojae*** **cross ACR10×P7076.**
(PDF)

Table S2 **Primer sequences.**
(PDF)
