# Supplementary material for: Genome Re-Sequencing and Functional Analysis Places the Phytophthora sojae Avirulence Genes Avr1c and Avr1a in a Tandem Repeat at a Single Locus
Source: PLoS One. 2014 Feb 24;9(2):e89738. doi: 10.1371/journal.pone.0089738 (PMC3933651; doi:10.1371/journal.pone.0089738)
Supplement: Figure S1 — Alignment of DNA sequences of Avr1c and Avr1a . (PDF) [file pone.0089738.s001.pdf]

Supplementary Figure S1. Alignment of DNA sequences of *Avr1c* and *Avr1a*

```
Avr1c_P6497      ATGCGCCTAACCAACACCCTCGTCGTGGCTGTTGCTGCCATCCTTCTCGCAAGCGAAAAC 60
Acr1c_P7074      ATGCGCCTAACCAACACCCTCGTCGTGGCTGTTGCTGCCATCCTTCTCGCAAGCGAAAAC 60
Avr1c_P7064      ATGCGCCTAACCAACACCCTCGTCGTGGCTGTTGCTGCCATCCTTCTCGCAAGCGAAAAC 60
Avr1a_P6497      ATGCGCCTAACCAACACCCTCGTCGTGGCTGTTGCTGCCATCCTTCTCGCAAGCGAAAAC 60
                 ************************************************************

Avr1c_P6497      GCTTTTTCTGCAGCAACTGATGCCGACCAGGCTACCGTCTCGAAGTTTGCGGCAGCGGAG 120
Acr1c_P7074      GCTTTTTCTGCAGCAACTGATGCCGACCAGGCTACCGTCTCGAAGTTTGCGGCAGCGGAG 120
Avr1c_P7064      GCTTTTTCTGCAGCAACTGATGCCGACCAGGCTACCGTTTCGAAGTTTGCGGCAGCGGAG 120
Avr1a_P6497      GCTTTTTCTGCAGCAACTGATGCCGACCAGGCTACCGTTTCGAAGTTGGCGGCAGCGGAG 120
                 ************************************** ******** ************

Avr1c_P6497      TTCGACACGTTGGTCGATGTCCTCACCACTGAGAGCAAGAGATCCCTTCGGGCCACGGTC 180
Acr1c_P7074      TTCGACACGTTGGTCGATGTCCTCACCACTGAGAGCAAGAGATCCCTTCGGGCCACGGTC 180
Avr1c_P7064      TTCGACACGTTGGTCGATGTCCTCACCACTGAGAGCAAGAGATCCCTTCGGGCCACGGTC 180
Avr1a_P6497      TTCGACACGTTGGTCGATGTCCTCACCACTGAGAGCAAGAGATCCCTTCGGGCCACGGTC 180
                 ************************************************************

Avr1c_P6497      GATGACGGCGAAGAGAGGTACAAGCTGTTTAAAATCGAAGCCTTGCAACAAGGAAAGTGG 240
Acr1c_P7074      GATGACGGCGAAGAGAGGTACAAGCAGTTTAAAATCGAAGCCTTGCAACAAGGAAAGTGG 240
Avr1c_P7064      GATGACGGCGAAGAGAGGTACAAGCTGTTTAAAATCGAAGCCTTGCAACAAGGAAAGTGG 240
Avr1a_P6497      GATGACGGCGAAGAGAGGTACAAGCAGTTTAAAATCGAAGCCTTGAAAAAAGGAAAGTGG 240
                 ************************* ******************* ** ***********

Avr1c_P6497      ACTTCATTTTTTAAAAAATGGAGCTCGAACGGCCGTACTGAGTCCGACATCAAGAAAAAG 300
Acr1c_P7074      ACTCCATTTTTTAAAAAATGGAGCTCGAACGGCCGTACTGAGTCCGACATCAAGAAAAAG 300
Avr1c_P7064      ACTTCATTTTTTAAAAAATGGAGCTCGAACGGCCGTACTGAGTCCGACATCAAGAAAAAG 300
Avr1a_P6497      ACGGATATTTTCAACAAATGGAAGGGTAACGAGCTTAGTCCGGCCGAGGTCCAGAACAAG 300
                 **     **** ** *******     ****  * ** *  * ****  ** **** ***

Avr1c_P6497      CTGGCGAATGTTAAATGGCTGTCCGA--ATCTGACAAAAGTAAGATTGTCGATAATTATG 358
Acr1c_P7074      CTGGCGAATGTTAAATGGCTGTCCGA--ATCTGACAAAGGAAGGCTTGTCGATAATTATG 358
Avr1c_P7064      CTGGCGAATGTTAAATGGCTGTCCGA--ATCTGACAAAGGAAGGCTTGTCGATAATTATG 358
Avr1a_P6497      CTGAAAAACAAGAAAT---TGAGCGACGATTTGAAAGACGCTA--TTTTCAGGAATTAT- 354
                 ***   **    ****   **  ***  ** *** * * *     ** **   ******

Avr1c_P6497      TATTCCACTTGAGAGGTCATTAG 381
Acr1c_P7074      TATTCCACTTGAGAGGTCATTAG 381
Avr1c_P7064      GATTCCACTTGAGAGGTCATTAG 381
Avr1a_P6497      -----------AAAGACTGGTAG 366
                            * **     ***
```
