# Supplementary material for: Genome Re-Sequencing and Functional Analysis Places the Phytophthora sojae Avirulence Genes Avr1c and Avr1a in a Tandem Repeat at a Single Locus
Source: PLoS One. 2014 Feb 24;9(2):e89738. doi: 10.1371/journal.pone.0089738 (PMC3933651; doi:10.1371/journal.pone.0089738)
Supplement: Table S1 — A list of 24 candidate Avh genes tested for co-segregation with Avr1c in the P. sojae cross ACR10×P7076. (PDF) [file pone.0089738.s002.pdf]

Supplementary Table S1. A list of 24 candidate *Avh* genes tested for co-segregation with *Avr1c* in the *P. sojae* cross ACR10 x P7076[1].

DNA marker	P7064 (Avirulent)			P7074 (Avirulent)			P7076 (Virulent)		
	INDEL	NA	AA	INDEL	NA	AA	INDEL	NA	AA
Avh1	0	18	15	0	20	16	-	-	-
Avh15	0	1	1	0	0	0	0	1	1
Avh20	2	0	0	0	2	2	0	2	2
Avh51	1	0	0	0	0	0	1	1	1
Avh62	0	0	0	0	0	0	0	26	21
Avh67	1	0	0	1	1	1	0	1	1
Avh71	0	0	0	0	4	2	1	3	1
Avh107	2	2	0	3	1	5	2	1	1
Avh161	0	0	0	0	0	0	0	0	0
Avh186	1	0	0	0	1	1	4	1	4
Avh187	1	0	0	0	1	1	4	1	4
Avh203	0	0	0	0	0	0	0	2	2
Avh225	-	-	-	0	0	0	3	2	14
Avh238	1	0	0	0	16	15	2	13	16
Avh263	0	1	1	1	3	1	1	1	1
Avh288	1	0	0	1	14	6	0	3	5
Avh307	1	0	0	5	73	51	3	59	43
Avh308	0	5	2	1	1	1	2	5	10
Avh320	0	12	10	0	0	0	0	16	12
Avh331	1	7	5	1	3	3	2	11	6
Avh343	0	0	0	0	0	0	0	0	0
Avh346	1	0	0	2	3	1	2	3	1
Avh362	1	0	0	0	0	0	0	4	3
Avh377	0	0	0	0	1	1	1	2	1

Polymorphisms in re-sequenced strains P7064, P7074, and P7076 are shown compared to the reference strain P6497, and virulence towards *Rps*1c is indicated. The reference strain P6497 is avirulent towards *Rps*1c. Number of nucleotide insertion/deletions (INDEL) or polymorphic sites (NA), or amino acid (AA) changes are shown.
