# Supplementary material for: Genome Re-Sequencing and Functional Analysis Places the Phytophthora sojae Avirulence Genes Avr1c and Avr1a in a Tandem Repeat at a Single Locus
Source: PLoS One. 2014 Feb 24;9(2):e89738. doi: 10.1371/journal.pone.0089738 (PMC3933651; doi:10.1371/journal.pone.0089738)
Supplement: Table S2 — Primer sequences. (PDF) [file pone.0089738.s003.pdf]

Supplementary Table S2. Primer sequences

Name	Marker Position Scaffold_7	Primer sequences	Restriction enzyme	Product Size (bp) ACR10	P7076	Application
1c poly69	91,241	GCAGCACTGCTTCAACTGC<br>TATTCGTTGCTGCGTGAAAG	BstNI	523	231+292	Mapping
1c poly73	1,028,642	GCAGCGACAAGAGCATTACG<br>GCTGGCTGTTGCTGCTGTT	BstNI	500	215+284	Mapping
1c poly74	1,446,220	AGGTCTTCGATTGTCGATCC<br>CTTGCCACTCATCGTCCAC	AlwN I	534	240+293	Mapping
1c poly76	1,611,925	AGATTCGTGGACTTGGTGCT<br>TGTTCAGTTGGTTCCCCATT	HpyCH4V	550	238+312	Mapping
1c poly59	1,641,992	GTCCCCCTGTAGTCCTTTGG<br>CACCGGATCATTGCAAACTT	Tsp509I	269+190	460	Mapping
1c poly29	1,673,008	GATCTACGGCCAAGACCAAG<br>GGATGGTTCGTCGTCTCCT	MspI	542	352+190	Mapping
1c poly8	1,675,636	CCAGCCTGAGAAGCAGTCTT<br>TGCAGAGCACTATGGAGCAT	Bsm I	255+344	599	Mapping
1c poly10	1,697,346	CCCTGGTCTGCGGAAATAC<br>CTGCTGGCAGAGGAGAGAAG	Rsa I	608	354+254	Mapping
1c poly113	1,827,846	GTGTTTGGCGGCTTCGAT<br>TGCAGCTCCGAATAGTGTTTT	BtsI	240+336	576	Mapping
1c poly110	2,046,577	CGTCCAACGGGTTAATAGACA<br>CCGCTGGAGCATGTATCTAA	BstBI	542	245+300	Mapping
1c poly118	2,328,857	TTGCAGCGATGTCTCATCC<br>TGCGGTACAGTAGGGAGAGG	BsiHKAI	102+274	376	Mapping
1c poly119	2,376,309	ACTTCCCCTACCGACTTGCT<br>GCCGGCCTTCTAAAGGTTTT	BsiHKAI	250+334	584	Mapping
1c poly92	2,682,606	GACGCCGAACTGAATACCTC<br>AGTTAACGTGGCTTCGTCGT	Dde I	600	234+321	Mapping
1c poly106	3,815,909	ACTTCACGATGAGCGCGTTA<br>TGCGACGACTCGAAAGGATA	Kpn I	562	241+321	Mapping
1c poly87	3,979,449	TGCAAGACGTTGGTAAGAACC<br>ACCCTGCGTCCTATCACCTA	HaeIII	247+318	565	Mapping
Avh275c pFF19F		GGCGGATCCATGGCAGCAACTGATGCCGAC				Cloning *Avr1C* alleles into biolistics construct pFF19
Avh275c pFF19R		GGAGCATGCCTAATGACCTCTCAAGTGGAATACATAATT				
Avh275c pFF19F		GGCGGATCCATGGCAGCAACTGATGCCGAC				Cloning *Avr1a* P6497 into biolistics construct pFF19
Avh275 pFF19R		GGAGCATGCCTACCAGTCTTTATAATTCCTGAAAATAGC				
Avh72pFF19F		GGCGGATCCATGCGACCACCAAGCGACAAT				
Avh275c R		CTAATGACCTCTCAAGTGGAATACATAATT				RT-PCR for detection of *Avr1c* expression
1a specific 1F		GGATATTTTCAACAAATGGAAGGGT				RT-PCR detection of *Avr1a* expression
1a specific 1R		CCGAAGTACCAGTCTACCAGTCTTT				
